# ^99m^Tc-labeled nanocolloid drugs: development methods

**DOI:** 10.1038/s41598-020-70991-2

**Published:** 2020-08-19

**Authors:** Vladimir Sadkin, Viktor Sкuridin, Evgeny Nesterov, Elena Stasyuk, Alexander Rogov, Natalya Varlamova, Roman Zelchan

**Affiliations:** 1 Tomsk Polytechnic University, 30 Lenina Avenue, 634050 Tomsk, Russia; 2 Tomsk National Research Medical Center, Russian Academy of Sciences, 5 Kooperativny street, 634050 Tomsk, Russia

**Keywords:** Cancer imaging, Drug development, Nanoparticle synthesis

## Abstract

The work considers the problem of obtaining nanocolloid radiopharmaceuticals (RPs) and studying their functional suitability for diagnosing sentinel lymph nodes (SLN) in cancer patients. Two principal approaches to the formation of technetium-99m-labeled particles based on inorganic and organic matrices were considered when carrying out research to develop methods for the production of nanocolloid RPs. The composition of the reagents and the conditions for obtaining nanocolloid radiopharmaceuticals were determined. The functional suitability of new RPs for scintigraphic diagnostics of sentinel lymph nodes has been studied.

## Introduction

**Figure 1:**
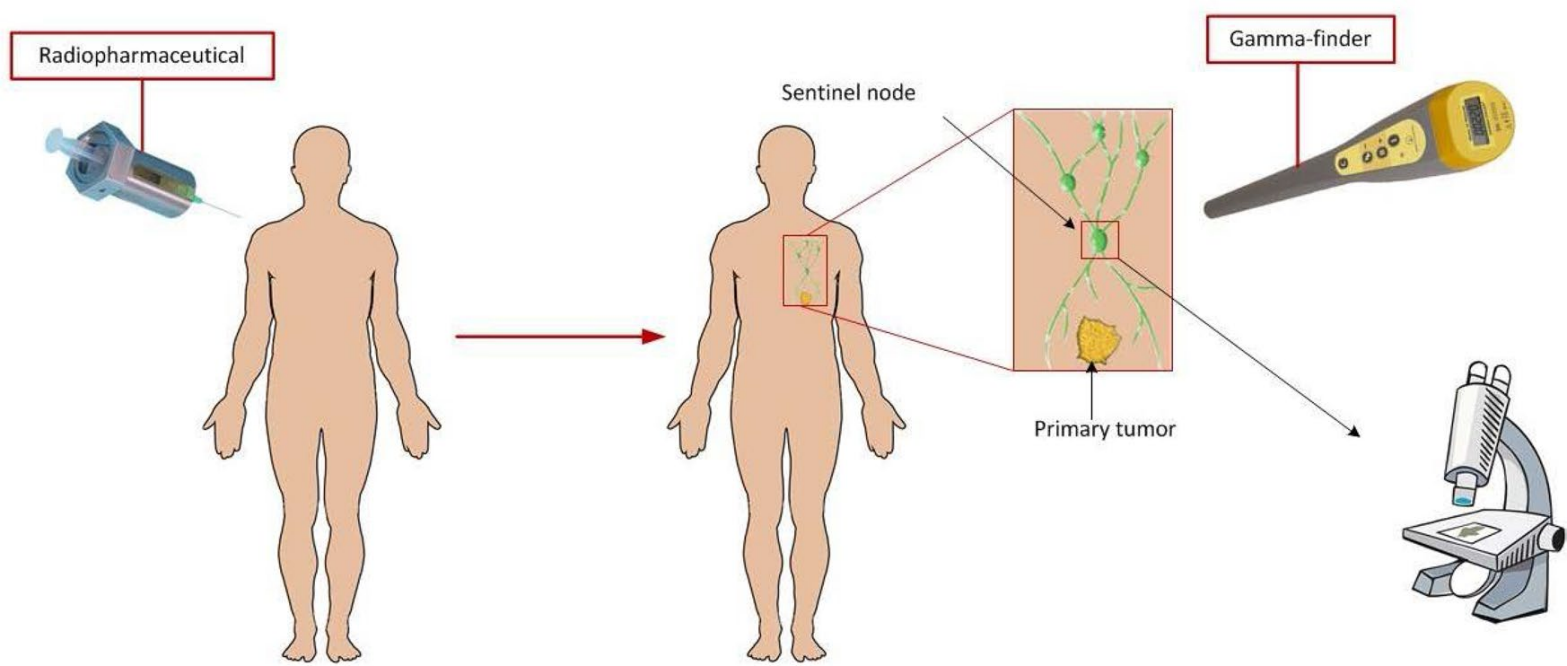
Scheme for determining the sentinel lymph node using nanocolloid radiopharmaceuticals (radiopharmaceutical, sentinel lymph node, detector).

The identification of sentinel lymph nodes—the first nodes that stand in the way of metastasizing of malignant neoplasms attracts increasing interest in modern oncological practice^1–5^. It is believed that if the SLN is not affected by the metastatic process, then all other regional lymph nodes are intact, so the results of biopsy of these nodes are an objective diagnostic criterion for the spread of malignant process (Fig. 1).

The optimal method of detecting SLN is the use of colloid nanomaterials labeled with technetium-99m for scintigraphic or radiometric determination of node localization^6–13^. Not so much the chemical nature of such particles but their size is the determining factor in the choice of the indicator in this case. Thus, according to Schauer^14^, a colloid with a particle size of less than 50 nm can accumulate not only in the SLN, but also at nodes of 2 and 3 orders of magnitude. Particles with the sizes of more than 100 nm slowly migrate from the injection site. The colloid with the particle size from 50 to 80 nm was recognized as the optimal one for detecting SLN.

The simplest method of obtaining colloids with given sizes and properties is immobilization of ^99m^Tc on the surface of nanosized materials.

Technetium-99m is by far the most popular radionuclide for conducting diagnostic studies, practically in all fields of medicine^15–18^. This is primarily due to its nuclear-physical characteristics: a relatively short half-life (6.02 h) and γ-radiation energy of 0.1405 meV, providing a low exposure dose and, at the same time, sufficient penetrating power for radiometric measurements.

Today, the Tc-99m Tilmanocept radiopharmaceutical is widely used, which has proven itself well and gives good results. But its production is quite time-consuming and requires expensive components. We offer a less laborious method from the simple components^19,20^.

## Materials and methods

### Materials

All the reagents were purchased from Sigma-Aldrich ACS grade and used without further purification. Technetium-99m was obtained from chromatographic ^99^Mo/^99m^Tc generator “^99m^Tc-GT-TOM” produced by Tomsk Polytechnic University (TPU)—Tomsk, Russia.

**Figure 2:**
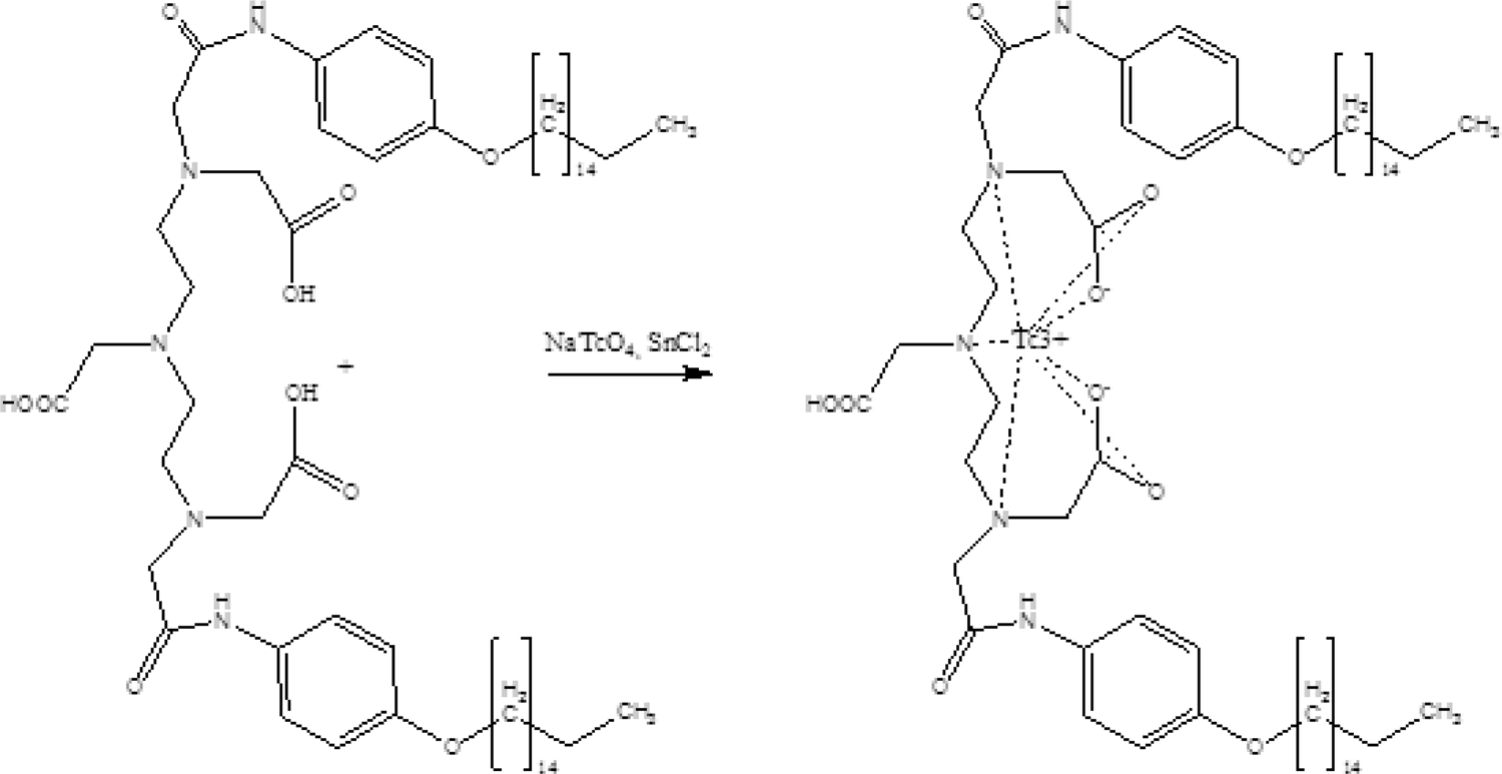
The general scheme for the synthesis of 99mTc-DTPA_mod_.

Three types of nanoparticles were selected to obtain nanocolloids labeled with ^99m^Tc. The first type of colloids was created on the basis of metal chelates with chemically modified complexons of diethylenetriamine-pentaacetic acid (DTPA). It should be noted that the DTPA molecule itself, like its complexes with metals, is hydrophilic and does not tend to form colloidal particles. The introduction of hydrophobic fragments into its structure allowed the preparation of water-insoluble modified DTPA complexes^21^. The original substance of the modified DTPA (DTPA_mod_) was synthesized in Tomsk Polytechnic University. Preparation of colloid solution DTPA_mod_ was produced using the following method. A sample of modified DTPA with the mass of 28 mg was quantitatively transferred to a volumetric flask of 50 ml and dissolved in 20 ml of 5% NaHCO_3_ solution by heating to 80 °C. After that, the volume was adjusted with the same solvent up to the mark. In order to reduce the particle size the container with suspension was heated in water to 70 °C and treated with ultrasound for 40 min, which reduced the average particle radius up to 55 nm. The general scheme for the synthesis of 99mTc-DTPA_mod_ is shown in Fig. 2.

**Figure 3:**
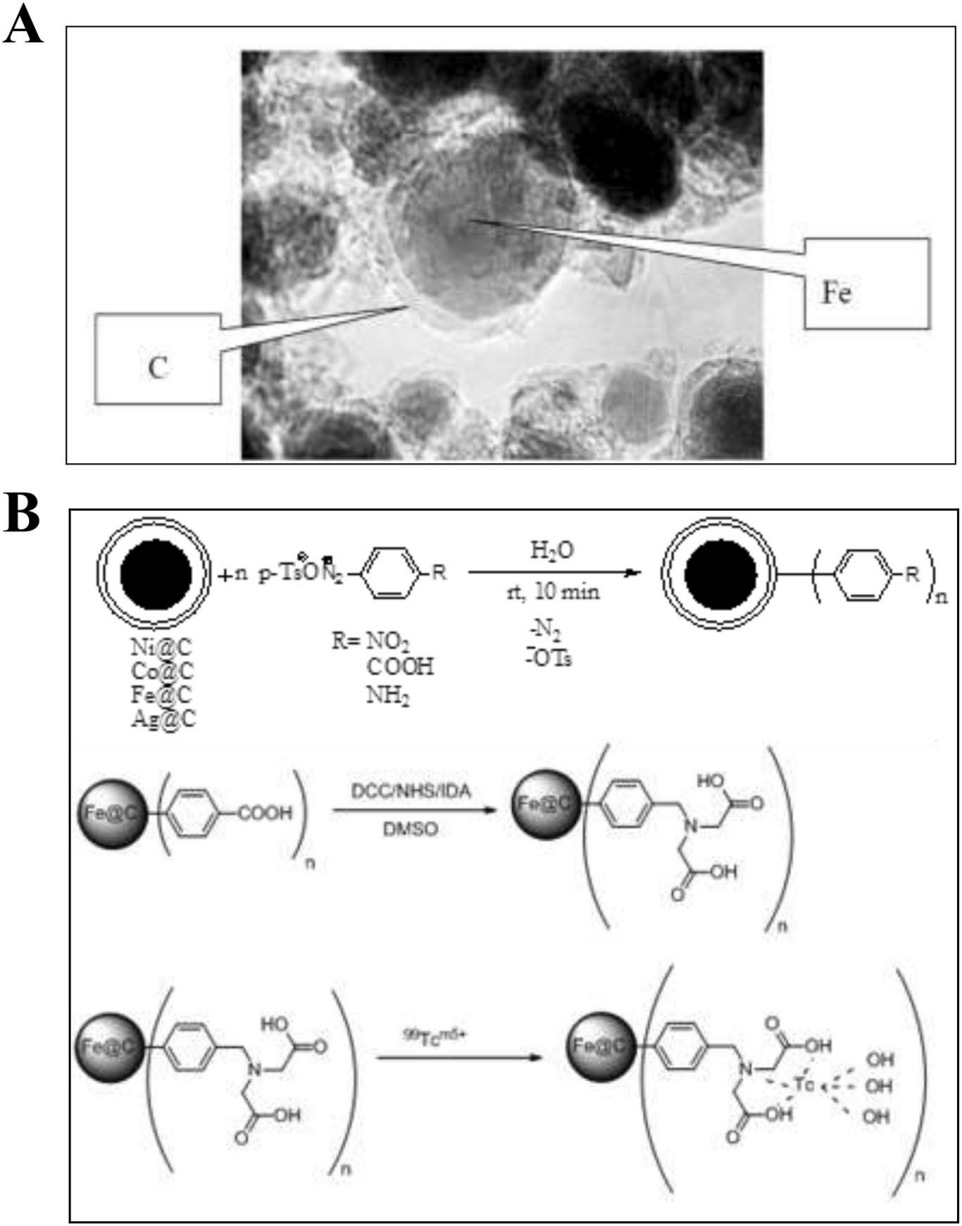
(**A**) Carbon encapsulated iron nanoparticle; (**B**) the general scheme for the synthesis of Fe@C particles.

The second type of colloids is iron nanoparticles coated with a carbon shell of Fe@C (Fig. 3a). These particles were obtained from the Institute of Metal Physics, UrB RAS (Ekaterinburg, Russia). In order to impart lipophilic properties to iron-carbon particles and to increase their stability in solution in the form of a colloid, a technique for preliminary deposition of organic radicals, aryldiazonium tosylates (ADT), onto the surface of these particles has been developed. An effective method for the synthesis of ADT followed by their application onto the carbon surface of particles was developed at the Tomsk Polytechnic University^22^. The general scheme for the synthesis of Fe@C particles and their subsequent interaction with 99mTc is shown in Fig. 3b.

In the third type of colloids technetium-99m was adsorbed on aluminum oxide powder. A powder of low-temperature (cubic) modification of gamma-oxide Al_2_O_3_, prepared from aluminum hydroxide powder Al(OH)_3_ by its calcination in a muffle furnace, was used. The substance was synthesized in Tomsk Polytechnic University.

A reducing agent—tin (II) chloride dihydrate was used in order to obtain complexes of ^99m^Tc with colloids.

Gelatin powdered, (Ph. Eur., USP-NF) pure, pharma grade. CAS Number: 9000-70-8 was purchased from (AppliChem GmbH (Darmstadt, Germany).

### Methods

#### Method for preparation of ^99m^Tc labeled nanocells

The introduction of the radioactive label ^99m^Tc into a colloidal substance was carried out by mixing of the selected substance with the reducing agent SnCl_2_∙2H_2_O (0.175–0.35 mg/ml) in different ratios and then adding a 4.0 ml of eluate of ^99m^Tc (280–500 MBq/ml) to the mixtures. The mixtures were incubated for 30 min at a temperature of 70–80 °C. The preparation is ready for use after cooling at room temperature. The reducing agent SnCl_2_∙2H_2_O was used as a hydrochloric acid solution. The amount of 0.07 g of tin chloride (II) is added to the vial and 0.2 ml of 1 M hydrochloric acid (HCl) is then added for its preparation. After dissolution, the volume is adjusted with distilled water to 10 ml. Dissolution was carried out in an inert gas (argon) medium.

#### Determination of the size of ^99m^Tc labeled colloidal particles

The determination of the size of the labeled nanocolloids was carried out by spectroscopy on a Nanophox particle size analyzer (“Sympatec GmbH”, Germany), and also by a technique, based on measuring the activity of the suspension before and after filtering it successively through filters with predetermined pore sizes: 220, 100, and 50 nm. Three samples were taken with a volume of 5 μl from each initial solution and filtrates for the subsequent measurement of their activity. Calculations of the yield of products with different particle sizes were determined according to the formulas given below:

$$C_{220} = \frac{{A_{vc} - A_{1} }}{{A_{vc} }};\,C_{100} = \frac{{A_{1} - A_{2} }}{{A_{1} }};\,C_{50} = \frac{{A_{2} - A_{3} }}{{A_{2} }},$$

where *A*_*vc*_ is the activity of the initial suspension prior to filtration; *A*_1_ is the activity measured after filtration through a 220 nm filter; *A*_2_ is the activity after filtration through 100 nm filter; *A*_3_ is the activity measured after filtration through 50 nm filter.

In parallel, determination of the radiochemical purity (RCP) of preparations by thin layer chromatography method was carried out.

#### Thin-layer chromatography (TLC) procedure

To determine radiochemical purity of ^99m^Tc–nanocolloid 5 µl of prepared sample was spotted on silica-gel impregnated strip (Sorbfil, Russia), 2 × 15 cm. To determine pertechnetate content of the radiopharmaceutical sample, first strip was developed using acetone as the mobile phase (time of chromatography 10 min). In this system, pertechnetate migrated with the front of the mobile phase (Rf = 0.9). To determine the colloid content of the preparations, the second strip was developed using ethanol:water:ammonium hydroxide (2:5:5) as the mobile phase (time of chromatography 40 min). In this system, the ^99m^Tc–nanocolloid migrated with the front of the mobile phase (Rf = 0.9)^23^.

#### Stability

The stability of ^99m^Tc–nanocolloid was studied in vitro by mixing of 5 ml of normal serum and 0.5 ml of ^99m^Tc–nanocolloid following by incubation at 37 °C for 8 h. At different time points (1 h, 4 h and 8 h) 0.2 ml aliquots of complex were removed and evaluated for radiochemical purity using TLC^24^.

#### Determination of the functional suitability of preparations for scintigraphic detection of SLN

A study to assess the functional suitability of new nanocolloid RPs was performed in 3 series of experiments involving 5 white Wistar male rats weighing 300–350 g. Injection of RP in a dose of 18–20 MBq was performed between the first and second fingers of the rat's hind paw. The animals were anesthetized with ether before the subcutaneous injection and during the scintigraphic study. Since the introduction, the kinetics of radiopharmaceutical distribution throughout organs and tissues was recorded by a frame-by-frame recording for 15 min (one frame—30 s) in a 64 × 64 pixel matrix. Static scintigraphy was performed after 1, 2, 3 and 24 h in the anterior and posterior projections in a matrix of 256 × 256 with a set of 500 pulses, scintigraphy of animals was performed on an E.CAM Signature 1800 gamma camera (Siemens, Germany). The results of scintigraphic studies determined the percentage of accumulation of RP in the lymph node and the injection site. The maintenance and participation of the animals in the experiment was carried out in accordance with the rules adopted by the “European Convention for the Protection of Vertebrates Used for Experiments or Other Scientific Purposes” (Strasbourg, 1986). The experimental protocols were approved by Cancer Research Institute Biomedical Ethics Committee, Protocol number 7/15. All invasive manipulations with animals were performed using inhalation or drug anesthesia.

#### Statistical analysis

All mean values are expressed as %ID/g ± SD. Data were analyzed statistically using methods of general statistics with a commercially available software package “Statistics for Windows” (StatSoft Inc., Version 6.0).

## Results and discussion

To carry out the labeling of colloids, ^99m^Tc, extracted from a standard generator in the form of pertechnetate ions contained in a 0.9% NaCl solution was used. It has a higher degree of oxidation (VII) in this chemical form and is not prone to complex formation. A reducing agent—tin (II) chloride dihydrate, widely used for the preparation of various compounds labeled with ^99m^Tc to was used to reduce its valence state, in order to obtain complexes with nanoscale particles^25^. As a result of the reaction of these components, the appearance of an un-targeted colloid is also possible due to the hydrolysis of excess SnCl_2_·2H_2_O or the additional formation of a complex of reduced ^99m^Tc with tin^26^. All this required preliminary experimental studies to establish the necessary and sufficient amount of SnCl_2_·2H_2_O in the reaction mixture.

During the experiments it was found that the optimal concentration of Sn (II) in the composition of the reaction mixture when it interacts with ^99m^Tc should be in the range of 0.175–0.35 mg/ml (Table 1).

**Table 1:** Change in relative activities of the complex [Sn-^99m^Tc] and ^99m^Tc (VII).

Amount of SnCl_2_∙2H_2_O, mg	A_[Sn-99mTc]_/A, %	A_Tc(VII)_/A, %
0.70	97.8	1.00
0.525	97.3	1.3
0.35	91.8	4.8
0.175	90.1	5.2

It was found that when the eluate with the preliminarily reduced ^99m^Tc (VII) was added to the nanoparticles (the Sn (II) concentration introduced in the RP was C_Sn_ = 0.02 mg/ml), almost the entire amount of ^99m^Tc has time to enter the composition of the large-size complex with tin even before its mixing with nanoparticles. This means that the sequence of the introduction and mixing of the reagents has a great influence on the labeling process, especially it concerns the introduction of the Sn (II) solution into the reaction mixture. In this connection, the reduction of ^99m^Tc with tin (II) was carried out in the presence of the selected substance. In this case, we can observe a "competitive" redistribution of the radionuclide between the substance and the tin complex. The technique of applying of the ^99m^Tc label to the surface of nanosized particles is given in the previous section.

As a result of the studies, reagent compositions and conditions for obtaining three nanocolloid RPs were determined. Table 2 shows their components, as well as the radiochemical purity and yield of the target colloid with particle sizes of 50–100 nm.

**Table 2:** Composition of reagents for production of technocium-99 m nanocolloids.

Composition of the preparation per 1 ml	RCP, %	Colloid yield 50–100 nm, %
DTPA_mod_ (1.0 mg) + ^99m^Tc (280–500 MBq) + SnCl_2_∙2H_2_O (0.35 mg) n = 5	94 ± 0.5%	< 60
Fe@C (1.0 mg) + ^99m^Tc (280–500 MBq) + SnCl_2_∙2H_2_O (0.175 mg) n = 5	96 ± 0.5%	< 40
Al_2_O_3_ (0.35 mg) + ^99m^Tc (280–500 MBq) + SnCl_2_∙2H_2_O (0.2 mg) n = 5	92 ± 0.5%	< 60

**Figure 4:**
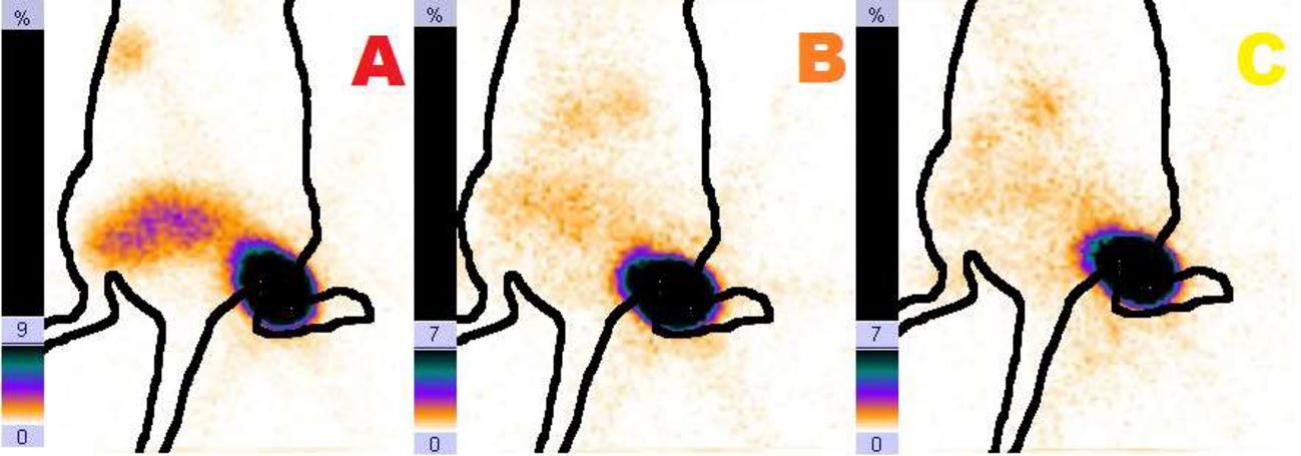
Distribution of the preparation in the rat when the preparation is administered: [Al2O3 + 99mTc + Sn (II)]: (**A**) immediately after the administration of the drug; (**B**) 30 min after the administration; (**C**) 60 min after the administration.

Proceeding from the chromatograms, it follows that the content of radiochemical impurity of unreduced ^99m^Tc (VII) in the obtained preparations is 2.7–3.5%. Preliminary tests of these preparations on experimental animals showed that accumulation in lymph nodes is practically not observed, although colloids have particle sizes in the required range from 50 to 100 nm. Scintigrams of rats obtained after subcutaneous administration of a technetium-99m labeled nanocolloid based on aluminum oxide are shown in Fig. 4.

Scintigrams showed that the drug remains at the injection point for 1 h without significant accumulation of ^99m^Tc in the blood of animals, which indicates a strong fixation of the radionuclide on the surface of the nanocolloid. Along with this positive point, it should be noted that accumulation of the preparation in the lymph nodes is not observed. Gelatin (G) was introduced into the reaction mixture in this connection, to increase the “mobility” of the labeled particles and increase the speed of their movement through the lymph system. Matrix systems based on gelatin provide a fairly uniform distribution of the immobilized substance and in this case, prevents the formation of a large tin colloid with ^99m^Tc. The results of the experiments showed that the addition of gelatin (2.5–4 mg/ml) to the reagent additionally provides an increase in the yield of the target colloid with particle sizes of 50–100 nm (Table 3).

**Table 3:** Indicators of RCP and the yield of a colloid with a fraction of 50–100 nm after the introduction of gelatin in the reagents.

Composition of preparations per 1 ml	RCP	Yield of colloid 50–100 nm, %
Al_2_O_3_ (0.35 mg) + ^99m^Tc (280–500 MBq) + SnCl_2_∙2H_2_O (0.2 mg) + G (2.5 mg) n = 5	95 ± 0.5%	< 76
DTPA_mod_ (1.0 mg) + ^99m^Tc (280–500 MBq) + SnCl_2_∙2H_2_O (0.35 mg) + G (2.5 mg) n = 5	95 ± 0.5%	< 89
Fe@C (1.0 mg) + ^99m^Tc (280–500 MBq) + SnCl_2_∙2H_2_O (0.175 mg) + G (2.5 mg) n = 5	94 ± 0.5%	< 97

**Figure 5:**
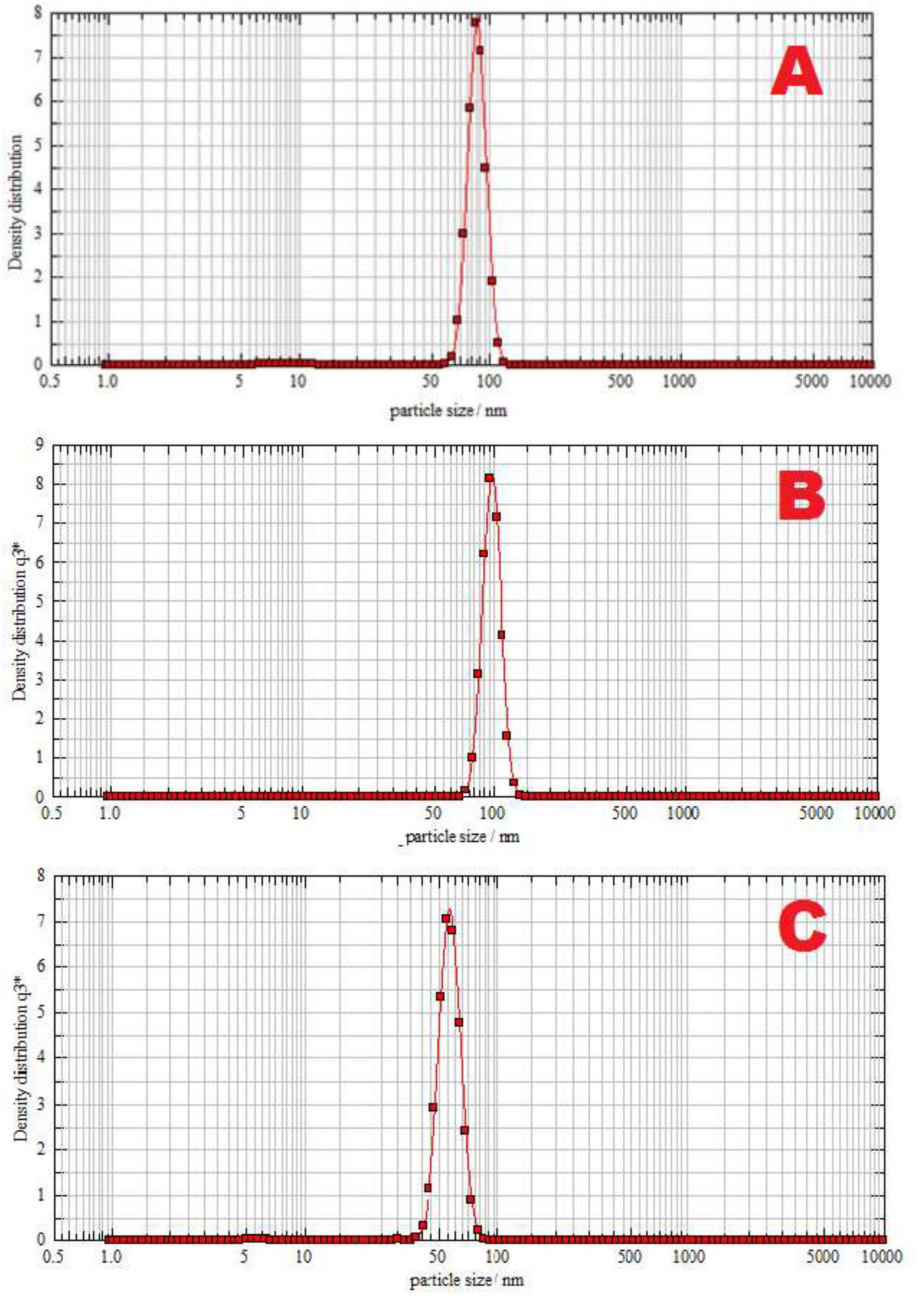
Change in the density of the distribution of the number of particles from their size in radiopharmaceuticals: (**A**) “99mTc-Al_2_O_3_”, (**B**) “99mTc-Fe@C”, (**C**) “99mTc-DTPAmod”.

In addition, the size of these particles was determined on a Nanophox particle analyzer. The obtained dependence of the change in the density of the distribution of the number of particles on their size, constructed from the results of a three-dimensional measurement of the preparations is shown in Fig. 5 (A, B, C). The average particle size (diameter) is 84, 92 and 55 nm, respectively.

Stability test showed that complex ^99m^Tc–nanocolloid was stable in normal serum at least for 6 h. Radiochemical purity of the tracer at the end of the experiment was 95 ± 0.5%.

**Figure 6:**
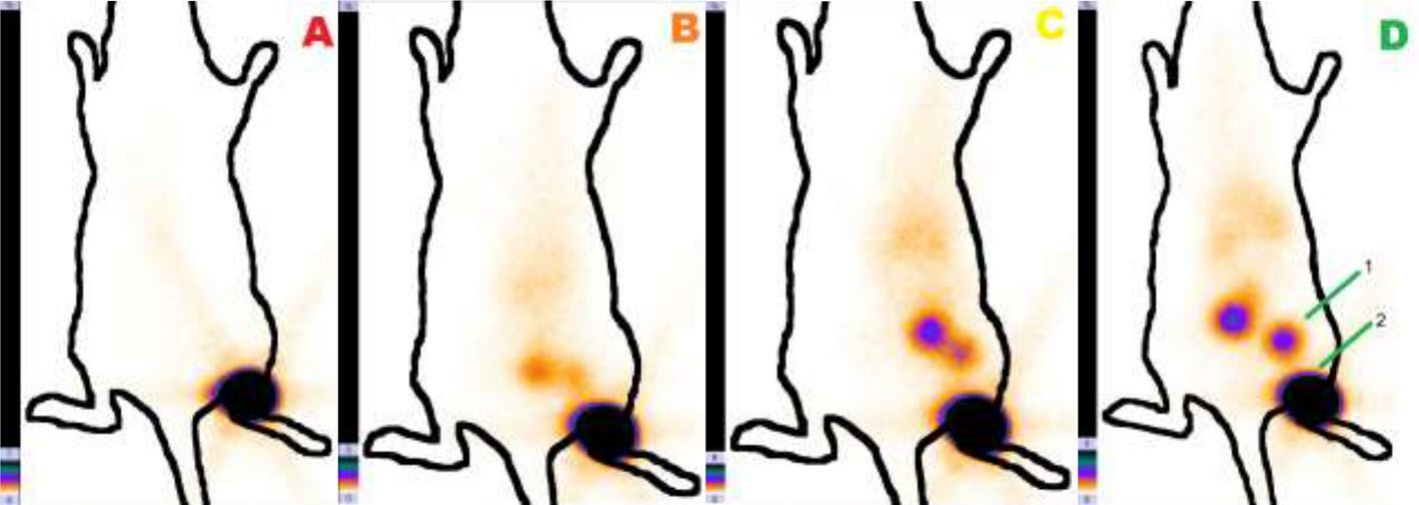
Distribution of the preparation in the rat with injection of suspension [Al_2_O_3_ + 99mTc + Sn (II) + Gelatin]: (**A**) immediately after the administration of the preparation; (**B**) 30 min after the administration; (**C**) 60 min after the administration; (**D**) 120 min after the administration. The numbers indicate: 1—lymph node, 2—site of preparation administration.

A study of the functional suitability of the obtained radioactive colloids for the scintigraphic imaging of the sentinel lymph nodes showed that these preparations provide a good level of accumulation in the sentinel lymph nodes (Fig. 6). Table 4 displays the Al_2_O_3_ + ^99m^Tc, DTPA_mod_ + ^99m^Tc, Fe@C + ^99m^Tc biodistribution data at different time points post-injection.

**Table 4:** Biodistribution data up to 2 h after injection of 18–20 MBq of ^99m^Tc in healthy male rats.

Time, h	Stomach		Liver		Spleen		Blood	Heart		Lungs	
	%	%/g	%	%/g	%	%/g	%/ml	%	%/g	%	%/g
^**99m**^**Tc-Al**_**2**_**O**_**3**_											
1	1.3 ± 0.2	0.48 ± 0.1	6.6 ± 0.3	0.55 ± 0.2	1.3 ± 0.2	0.65 ± 0.1	0.2 ± 0.1	0.3 ± 0.1	0.25 ± 0.09	0.6 ± 0.1	0.22 ± 0.09
2	1.7 ± 0.2	0.61 ± 0.15	10.5 ± 0.9	0.87 ± 0.21	2.1 ± 0.3	1.1 ± 0.15	0.24 ± 0.04	0.3 ± 0.06	0.24 ± 0.008	0.6 ± 0.12	0.22 ± 0.1
3	1.8 ± 0.4	0.62 ± 0.22	13.5 ± 2.07	1.15 ± 0.24	2.8 ± 0.5	1.4 ± 0.14	0.21 ± 0.03	0.4 ± 0.09	0.25 ± 0.008	0.51 ± 0.15	0.23 ± 0.08
5	1.8 ± 0.7	0.63 ± 0.31	17.3 ± 1.9	1.35 ± 0.31	3.5 ± 0.7	1.65 ± 0.2	0.27 ± 0.07	0.3 ± 0.11	0.29 ± 0.06	0.59 ± 0.14	0.31 ± 0.06
24	2.4 ± 0.4	0.87 ± 0.38	18.3 ± 1.4	1.52 ± 0.29	3.6 ± 0.6	1.80 ± 0.2	0.3 ± 0.1	0.4 ± 0.15	0.23 ± 0.01	0.54 ± 0.2	0.20 ± 0.1
^**99m**^**Tc-DTPA**_**mod**_											
1	1.2 ± 0.2	0.48 ± 0.1	6.6 ± 0.3	0.55 ± 0.2	1.3 ± 0.2	0.65 ± 0.1	0.2 ± 0.1	0.3 ± 0.1	0.25 ± 0.09	0.6 ± 0.1	0.22 ± 0.09
2	1.5 ± 0.2	0.60 ± 0.15	10.5 ± 0.9	0.87 ± 0.21	2.1 ± 0.3	1.1 ± 0.15	0.24 ± 0.04	0.3 ± 0.06	0.24 ± 0.008	0.6 ± 0.12	0.22 ± 0.1
3	1.5 ± 0.4	0.62 ± 0.22	13.9 ± 2.07	1.15 ± 0.24	2.8 ± 0.5	1.4 ± 0.14	0.27 ± 0.03	0.31 ± 0.09	0.25 ± 0.008	0.61 ± 0.15	0.23 ± 0.08
5	1.5 ± 0.7	0.63 ± 0.31	16.3 ± 1.9	1.35 ± 0.31	3.3 ± 0.7	1.65 ± 0.2	0.27 ± 0.07	0.3 ± 0.11	0.24 ± 0.06	0.59 ± 0.14	0.21 ± 0.06
24	2.2 ± 0.4	0.88 ± 0.38	18.3 ± 1.4	1.52 ± 0.29	3.6 ± 0.6	1.80 ± 0.2	0.3 ± 0.1	0.4 ± 0.15	0.23 ± 0.01	0.54 ± 0.2	0.20 ± 0.1
^**99m**^**Tc-Fe@C**											
1	1.1 ± 0.2	0.48 ± 0.1	6.6 ± 0.3	0.55 ± 0.2	1.3 ± 0.2	0.65 ± 0.1	0.2 ± 0.1	0.3 ± 0.1	0.25 ± 0.09	0.6 ± 0.1	0.22 ± 0.09
2	1.6 ± 0.2	0.60 ± 0.15	10.5 ± 0.9	0.81 ± 0.21	2.2 ± 0.3	1.1 ± 0.15	0.24 ± 0.04	0.3 ± 0.06	0.24 ± 0.008	0.6 ± 0.12	0.22 ± 0.1
3	1.4 ± 0.4	0.62 ± 0.22	13.9 ± 2.07	1.15 ± 0.24	2.8 ± 0.5	1.6 ± 0.14	0.27 ± 0.03	0.31 ± 0.09	0.25 ± 0.008	0.61 ± 0.15	0.25 ± 0.08
5	1.7 ± 0.7	0.63 ± 0.31	15.3 ± 1.9	1.35 ± 0.31	3.3 ± 0.7	1.65 ± 0.2	0.27 ± 0.07	0.3 ± 0.11	0.24 ± 0.06	0.59 ± 0.14	0.21 ± 0.06
24	2.6 ± 0.4	0.88 ± 0.38	18.7 ± 1.4	1.52 ± 0.29	3.6 ± 0.6	1.80 ± 0.2	0.4 ± 0.1	0.6 ± 0.15	0.23 ± 0.01	0.54 ± 0.2	0.20 ± 0.1

Data represent the average value (n = 5).

The level of accumulation of preparations in the lymph nodes is 1.8–5.4% of the total injected activity.

## Conclusion

As a result of the studies, the composition of the reagents and the conditions for the synthesis of three nanocolloid RPs were determined. An experimental dependence of the change in the content of ^99m^Tc (VII) impurities on the concentration of tin (II) was established and its minimum amount (0.175 mg/ml) was determined to reach a RHP greater than 94%. In this case, the yield of the target colloid with particle sizes of 50 ± 100 nm is 42–76%. Preliminary tests of the developed preparations on experimental animals showed that accumulation of RP in lymph nodes is practically not observed, although the sizes of colloidal particles are in the required range. Increase in the speed of "transportation" of colloids through the lymphatic system was achieved by the introduction of gelatin in the composition (2.5–4 mg/ml). In addition, there was an increase in the yield of the colloid with particle sizes of 50–100 nm to 76–97% with radiochemical purity of the preparations of 94–95%. Repeated studies in experimental animals have shown that all synthesized nanocolloid preparations provide a good level of accumulation in the SLN. Thus, the level of accumulation of RP “^99m^Tc-DTPAmod” and RP “^99m^Tc-Fe@C(ADT)” in the SLN is 3.6% and 3.8%, respectively. At the same time, the accumulation level of the preparation based on aluminum oxide is 5.4% of the total input activity.
